# Discussions on Dental Pathology and Surgery

**Published:** 1870-12

**Authors:** Thomas O. Summers


### DISCUSSIONS ON DENTAL PATHOLOGY AND SURGERY.

REPORTED BY THOMAS O. SUMMERS, A. M.

After the reading of his report, Dr. Atkinson continued as follows, illustrating his subject by well executed diagrams:

That I may be clean, I feel bound to say to you, that not a word in that report is mine, but it is taken from the fount of inspiration which wells up within me. When it is fitting you'll get the rest. Are you able to bear the truth? You know what our Savior said to Zebedee's children, "Are ye able to drink of the cup that I shall drink of, and be baptized with the baptism that I am baptized with?" Like all fools, they answered "We are able." Now I am not so far gone as that. Remember I am speaking to your hearts and consciences, as well as your intellects. I am going to vindicate the great Architect of the Universe.

We have, 1. Dermatozoa. They have simply skin and contents; endowed only with touch. They have feeling only. Their nature is savage. Their idea of divinity is power, *force.* Their marital relation is "touch and go"—indiscriminate. 2. Next we have articulata, which have tongues. To touch they add taste. They associate by two senses. Nature, barbarous; custom, clanish. Reigning religious idea, *fear.* Marital relation, *inconstant.* 3. Vertebrates. This is not brought about by cataclysm, but is a serial development—the rocks are as human as you. This class is the Rhinozoa. Three senses—touch, taste, and smell. Semi-civilized. Political idea, a republic. Religious idea, *Jehovah.* Force and fear are the reigning elements. There our friends, the Jews, stand to-day. Marital relation, polygamy. 4. Ottozoa. These have four senses—touch, taste, smell and hearing. Their political idea is democratic and republican. Religious idea, Paternism—God the Father.

Marital relation, monogamy. 5. Ophthalmozoa. They have five senses. They add sight to all the rest. Here music and color come in. Political idea, republic. Religious idea, Immanuel—God with us. In their marital relations, their affections are ahead of their intellects. Here conjugiality is the leading idea. Marital slavery is swept away by the besom of destruction and love, for the Lord is with you.

That means health of mind, body, soul and spirit. Its *ghost*. La Place, said, "he had dissected many and many a subject that he might find the soul." Why he is not begotten yet, let alone gestated and brought forth. We turn from the light and say it is as dark as hell. Now we have three foundations on which to build. 1. Crystalosophy. They say crystal is dead, that they belong to the Azoic period, which was perfected before the vegetable. 2. Cellosophy. 3. Corpusculosophy. This last has nitrogen in it.

We have gone slowly in medicine, simply because we have been dominated by past stages of development and processes of destruction. We have given credit to teachers for knowing more than they really do. Now you have heard me laud the saturated solution of the chloride of zinc, used to stop necrosed action in bone.

Now when I say I have something of a *new love*, you will say I am not consistent with the doctrine I teach—that I am not a *monogamist*.

But I deny this charge.

Now, sulphuric acid is the remedy for necrosis or caries of bone, from the chemically pure down to the compound aromatic sulphuric acid—the elixor vitriol, ($SO_3$,HO) of the shops. If I had heard that twenty years ago, I would not have believed it. It is based on the law of affinities in that diagram which I have just explained. I once had a case of necrosed spinous process of the second lumbar vertebra, in a female patient. By inspiration, for you know it is the inspiration of the Almighty that giveth us understanding, I

touched it with sulphuric acid, and I had such a cure, that the surgeons denied that there had ever been any necrosis at all. I acted on that hint, and am now ready to pronounce it a *cure*. There is an affinity between the salts of lime which make the bone hard, and the sulphuric acid, (when the bone is deprived of its vitality), that it acts on all the lime deprived of animal life, and produces solution. The following expresses the reaction:

Phosphate of lime + sulphuric acid = sul. lime + hyd. phos. ac. $CaO\ PO_5 + SO_3\ HO, = CaO, SO_3 + PO_5\ HO$. Sulphuric acid does not act on cell life. I have had such marvelous results from this treatment, that I announce it from my head clear down to my *pyramidalis abdominalis*."

If you follow *this*, ugly cutting in the mouth is at an end. What are the forms of diseased bone to be treated in this way? One is when the solution of lime salts leave the matrix of animal matter—*mollities ossium*. Another form of death in bone is necrosis, where a considerable territory is deprived of pabulum. Another, osteal ulceration or caries, where only the outside stratum of cells is thus affected, and there is an attempt at reproducing the solution of lime salts. In this case there is considerable swelling and drawing up like the *columnæ carnæ* of the heart, and you will diagnose it as *osteal cyst*. It is not *exostosis*. Now what do you do? Why puncture it and inject sulphuric acid. Protect the external parts. Make a little pocket so as to form a clot. With a little ingenuity you can accomplish this with a bit of rubber. Now don't go and inject three quarts in a person's mouth. Clean out the cavity, and get it as dry as possible with bibulous paper. Then take a hypodermic syringe and inject the sulphuric acid, drop by drop, and it will be drunk up by the necrosed bone like syllabub. Then dress it with two parts tannin and one part glycerin on a little mat of cotton.

This treatment will do for any bone that is necrosed.

You can discover whether the bone is saturated in two or three days, or eight days at most.

To speak of the differentiation between the saturated solution of the chloride of zinc, and sulphuric acid. The latter has not so penetrating and multiplied extent of action. When the chloride of zinc is used, the capillaries collapse, and the blood regurgitates, and this goes on as far as the next switch of the capillaries with the venous and arterial radicals, and forms of the capillaries as far as the affinity is satisfied, the hydro-chlor. zincate of albumen, and where it is only strong enough to act as an astringent the reflow of the blood column will re-open the capillary and admit the fluid blood, and the red and white corpuscles, and the nutrient action is then established after its normal mode. It creates a foreign body. What becomes of this?

Dr. Carroll: "It is expelled."

Dr. Atkinson: "What does nature do with foreign bodies?"

Dr. Peebles: "It first dissolves them, then they pass through the tissues in the form of gas."

Dr. Atkinson: "That's nothing but expulsion. Can any one answer the question?"

Dr. Morgan: "Nature rids herself of foreign bodies in two ways. 1st. By direct expulsion. 2d. By encysting them."

Dr. Atkinson: "That's right. Light has broken in on us all of a sudden. Now it is marvelous the toleration of interference of the superior maxillary bone. If this had not been the case it would have been destroyed long ago, for it has received such treatment at the hands of Dentists, as equals the horrors of Pandemonium itself. We have found out that nature either encysts or expels a foreign body. Now this depends on temperament, temperature, exercise, diet, light, shade and many other things which are a part of your life. Sulphuric acid kills all mineral life as this is a mineral acid, and the bone is renewed after its original pattern. I am sure the time is coming when we shall be able to redeem every diseased molecule, and know the processes of its original formation.

When the necrosed portion of bone has been sufficiently saturated, then wash out the cavity, and apply a little tincture of calendula. If there is an abscess which is not open, you can open it with a dressing needle, wash out the cavity, dry it with bibulous paper, and then put the sulphuric acid in it, and be sure you are not doing it for money, and you'll succeed."

A member: "What strength of sulphuric acid would you prefer?"

Dr. Atkinson: "That most preferable is pure sulphuric acid, when the disease is well defined. When this is not the case, use 1 part water, and 1 part sulphuric acid."

